# Diagnosis and Staging of Necrotizing Enterocolitis: Current Controversies and a Phenotype-Based Framework

**DOI:** 10.3390/children13060758

**Published:** 2026-05-29

**Authors:** Usha Devi, Jörn-Hendrik Weitkamp, Jeffrey S. Shenberger, Parvesh Mohan Garg

**Affiliations:** 1 Department of Neonatology, Jawaharlal Institute of Postgraduate Medical Education Research, Pondicherry 605006, India; jd1356@jipmer.ac.in; 2 Department of Pediatrics/Neonatology, Monroe Carell Jr. Children’s Hospital at Vanderbilt, Nashville, TN 37232, USA; hendrik.weitkamp@vumc.org; 3 Department of Pediatrics/Neonatology, Connecticut Children’s, Hartford, CT 06106, USA; jshenberger@connecticutchildrens.org; 4 Department of Pediatrics/Neonatology, Atrium Health Wake Forest Baptist, Wake Forest School of Medicine, Winston Salem, NC 27157, USA

**Keywords:** enterocolitis, necrotizing, diagnostic criteria, phenotypes, infant, premature, dysbiosis, intestinal ischemia

## Abstract

**Highlights:**

**What are the main findings?**

- Necrotizing enterocolitis (NEC) is a heterogeneous syndrome rather than a single disease, with multiple distinct clinical phenotypes caused by different pathophysiological mechanisms.
- The traditional staging system has significant limitations as a diagnostic tool due to nonspecific criteria, non-linear progression, and overlap with clinical mimickers.

**What is the implication of the main finding?**

- A phenotype-based, dynamic diagnostic framework can improve diagnostic precision, reduce misclassification, and better distinguish NEC from its mimics.
- Shifting from stage-based labeling to phenotype-informed approaches may enhance clinical decision-making, research design, and the development of targeted therapies.

**Abstract:**

Necrotizing enterocolitis (NEC) remains one of the most devastating gastrointestinal emergencies in neonates and also presents major diagnostic challenges. Despite extensive research, NEC still lacks a practical definition and relies on a set of nonspecific clinical, laboratory, and radiological findings rather than a single pathognomonic presentation or test. The modified Bell staging system remains the most widely used framework in clinical practice and research, but it was originally developed to guide treatment decisions rather than aid diagnosis and has important limitations when applied as a diagnostic aid. Clinical and radiological criteria used for early stages of NEC are nonspecific, disease progression is not always linear, radiographic signs are inconsistently present, and histopathological confirmation is unavailable in most of the cases as surgery is not undertaken in all the cases. These limitations have led to the opinion that even the modified Bell staging is “broken” when it is used to define the disease itself. At the same time, increased understanding about gut immunity and microbiome progression, and neonatal hemodynamics make it increasingly clear that NEC is not a single uniform disease. It is now regarded as a heterogeneous syndrome comprising multiple phenotypes that share a final common pathway of intestinal injury and necrosis differing in timing, predisposing factors, mechanism, and clinical course. These presentations overlap with several neonatal conditions including spontaneous intestinal perforation, septic ileus, cow’s milk protein allergy, congenital heart disease-related intestinal hypoperfusion, viral enterocolitis, malrotation with volvulus, and intussusception. This review discusses controversies in the definition and staging of NEC, consolidates alternative diagnostic criteria beyond Bell’s system, and elaborates on a phenotype-based framework for clinical distinction. Also, the review sheds light on the clinical mimickers, practical bedside diagnosis using serial clinical assessment and imaging, consequences of NEC, and emerging precision medicine approaches. A shift from stage-based labeling toward a practical, phenotype-informed framework may improve diagnostic precision, reduce misclassification, and enhance both clinical care and research.

## 1. Introduction

Necrotizing enterocolitis (NEC) is a multifactorial inflammatory disease resulting in necrosis of the neonatal intestine. It primarily affects preterm and very-low-birth-weight (VLBW) infants and remains one of the devastating diseases in neonatal intensive care [1,2]. Although survival of extremely preterm infants has improved globally, NEC continues to contribute to substantial mortality, surgical burden, and long-term morbidity. Though population incidence varies with the definition used and population studied, among VLBW infants it is commonly between 5% and 10%, with highest burden among those born at the lowest gestations [2,3]. Mortality is high around 30–50%, especially when surgical intervention is needed or when disease is fulminant [3,4].

The impact of NEC on the survivors extends beyond the acute episode. Survivors may develop intestinal strictures, short bowel syndrome, intestinal failure, prolonged need for parenteral nutrition, cholestatic liver disease, postnatal growth failure, repeated rehospitalization, and adverse neurodevelopmental outcomes [5,6,7,8]. Severe NEC also imposes a major emotional and financial burden on families and health systems. In total, these consequences make the precise diagnosis important for more than academic purposes, appreciating its influence on bedside decisions, epidemiology, trial design, and long-term counseling.

Defining and diagnosing NEC with precision is very difficult. There is no single biomarker, universally reliable imaging finding, or single clinical feature that is present in all cases. Instead, diagnosis is made from combinations of abdominal signs, systemic instability, laboratory abnormalities, and imaging findings. Many of these findings are common in premature infants with other illnesses such as septic ileus, feeding intolerance, or spontaneous intestinal perforation [9,10,11]. Due to the overlapping features between these conditions, both overdiagnosis and underdiagnosis of NEC can occur.

The modified Bell staging system is mostly commonly used for NEC diagnosis and staging both in clinical and research setting [12,13]. Bell’s original work offers a practical framework linking the severity of illness to treatment decisions. Over time, however, this staging system was increasingly used as a surrogate diagnostic definition, even though it had not been designed for that purpose. In particular, “Stage I NEC” often includes neonates with nonspecific gastrointestinal and systemic abnormalities who would neither develop nor progress to have intestinal necrosis [9,10]. Moreover, progression is not in the orderly stage-wise manner as given in Bell staging. Some infants present abruptly with fulminant disease, while others improve without progression.

Our biological understanding of NEC has evolved dramatically. NEC is now recognized as a multifactorial syndrome arising from interactions among intestinal immaturity, microbial dysbiosis, exaggerated inflammation, epithelial injury, impaired barrier function, and in some infants, mesenteric hypoperfusion or ischemia-reperfusion injury [14,15,16,17,18,19,20]. This evolving understanding necessitates disease paradigm that accommodates heterogeneity. Accordingly, many alternative definitions, consensus case criteria, and phenotype-based approaches that separate “suspected intestinal disease” from “definite NEC” have been developed to distinguish NEC from its mimickers. Such definitions also recognize that different clinical phenotypes may represent biologically distinct forms of intestinal injury [10,11,21]. In this review, we will discuss why Bell staging is inadequate as a diagnostic framework. We then discuss alternative definitions and the rationale for treating NEC as a syndrome composed of overlapping phenotypes. We expand upon these phenotypes in detail, discussing differential diagnoses and mimickers, and propose a dynamic, integrative approach to diagnosis that is more clinically actionable.

## 2. The “Broken Bell”: Rethinking Traditional Bell Staging

The modified Bell staging system is universally employed because of the simplicity, yet several of its core assumptions limit its usefulness as a diagnostic framework today [9,10,12,13]. The first major problem is the non-specificity of early-stage criteria. Bell Stage I includes increased gastric residuals, abdominal distension, vomiting, occult or gross blood in stool, apnea, bradycardia, temperature instability, and intestinal dilatation or ileus [13]. All these features can occur in premature infants for reasons other than NEC. Infection, noninvasive ventilation-related abdominal distension, feed intolerance, postoperative ileus or viral infection can produce a similar picture. Thus, labelling an infant as having Stage I NEC often reflects diagnostic uncertainty.

The next problem is the staging suggestive of linear progression. Bell staging suggests that the disease progresses from suspected NEC to definite NEC to advanced disease. In day-to-day practice, however, NEC progression is not reliably linear. Some infants are first recognized in advanced disease when pneumoperitoneum or profound shock is already present. Few infants oscillate between mild and moderate signs; still, others are treated for “possible NEC” and later labelled as not having NEC at all [9,10].

**Figure 1:**
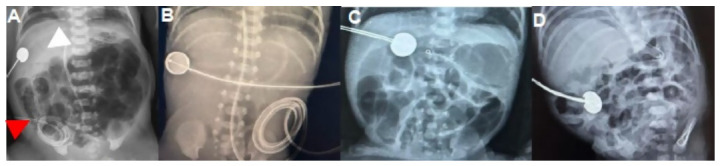
(**A**) Classical radiographic features in a neonate with NEC: pneumatosis intestinalis (red arrowhead) and portal venous gas (white arrowhead)—stage 2B according to modified Bell staging. (**B**) Radiograph showing gasless abdomen in a 27-week preterm infant with altered gastric aspirates, a tense abdomen, abdominal discoloration, and a clinical picture of NEC. (**C**) Clinically suspected and histopathologically proven NEC with radiograph showing dilated bowel loops alone. (**D**) Radiograph of a 28-week preterm infant with recurrent episodes of apnea and soft abdomen, yet showing pneumatosis. The repeat X-ray within 24 h was normal, and the infant was clinically well.

A third major limitation relates to imaging findings. The pathognomonic radiographic sign of NEC, pneumatosis intestinalis, is highly useful when clearly present, but it is not always seen [22,23]. It may be transient, absent in some severe cases, or difficult to distinguish from stool or intraluminal gas. Portal venous gas may also be fleeting. In extremely premature infants, radiographs may show only nonspecific bowel dilatation, fixed loops, or a gasless abdomen. Inter-observer variability in interpretation is well documented, and agreement is often poorest especially in the cases where there is diagnostic dilemma [22]. Consequently, criteria that rely heavily on classical radiographic signs may lack sensitivity, whereas those that do not consider imaging requirements lose specificity (Figure 1).

The fourth limitation is that Bell staging is often used in a manner that mixes diagnosis and severity stratification. A diagnostic definition should identify whether the disease is present; a severity system should estimate the advanced nature of the disease. Both are attempted simultaneously using the Bell staging. This can be problematic because an infant may be severely ill due to volvulus or spontaneous intestinal perforation (SIP) and not NEC, while another may have genuine NEC without characteristic signs [11,24]. The conceptual mixing of diagnosis and severity contributes to inconsistent case classification.

The fifth limitation is the rarity of histopathological confirmation. In NEC, pathological diagnosis is generally available only in infants who undergo surgical resection bowel or occasionally at autopsy. The findings commonly vary from patchy mucosal necrosis to diffuse transmural necrosis with perforation. Some surgical conditions initially labeled as NEC may later appear more consistent with isolated perforation or another etiology [4,24]. Thus, the pathological “gold standard” is not always available for most cases and findings are also not uniform.

Finally, Bell staging was devised prior to deeper knowledge of the roles of intestinal epithelial signaling, Toll-like receptor 4 activation, microbiome disruption, human milk protection, inflammatory priming, and hemodynamic vulnerability in growth-restricted or cardiac infants in pathogenesis [14,15,16,17,18,19,20]. Since the staging system is based on severity rather than mechanism involved, it is less useful for distinguishing distinct biological pathways presenting with similar bedside signs.

These concerns have led some authors to describe Bell’s framework as “broken” when used as a disease definition [9]. A revised framework should ideally accomplish several things: separate certainty of diagnosis from severity, improve distinction from NEC and its mimickers, acknowledge gestational-age-specific variable presentations, incorporate newer imaging modalities, and accommodate clinical phenotypes rather than attributing all presentations to a single linear pathway.

## 3. Alternative Diagnostic Criteria

Recognition of the limitations of Bell staging has led to repeated attempts to define NEC more rigorously so as to improve comparability across studies and reduce the erroneous inclusion of infants who do not actually have NEC [9,10,11,21,25]. The development of consensus-based definitions is intended for research use as well. They are based on a combination of clinical deterioration and other evidence of intestinal disease, such as characteristic imaging, surgical findings, or pathology, and attempt to exclude NEC mimickers like isolated spontaneous intestinal perforation [11,21]. Another important development is gestational-age-specific case definitions. Battersby and colleagues argued that NEC does not present similarly across different gestational ages and proposed a gestational age specific definition intended to improve diagnostic consistency, particularly in very preterm infants [10]. This work highlighted that highly immature infants may show less classic radiographic findings and more systemic instability, whereas more mature infants may exhibit classic features on imaging or more specific intestinal signs. A “one size fits all” definition may therefore perform poorly at the edges of prematurity.

Some proposed frameworks also distinguish between “suspected,” “probable,” and “definite” NEC. This takes into account how diagnosis changes at the bedside. The clinician’s diagnostic certainty changes over time as serial examinations, radiographs, ultrasound, laboratory trends, and response to treatment evolves. Instead of only a binary label, probabilistic models acknowledge uncertainty while also clearly expressing the clinical concern.

The Vermont Oxford Network and other registries have also employed operational definitions for reporting and benchmarking [25]. Although these definitions increase standardization, they have challenges including heavy dependence on documentation and imaging interpretation. Apparent differences in NEC rates between individual care locations not only reflect the care practices and outcomes but also depend on the case definition used. The trade-off between sensitivity and specificity lies at the heart of NEC diagnosis. If the threshold for diagnosis is lowered, more infants are treated and research cohorts become diluted. If it is raised, some early disease may be missed.

Another critical area requiring attention is the separation of NEC from SIP, which differs in timing, pathogenesis, pathology, radiology, and risk factor profile. It usually presents earlier, often without diffuse bowel inflammation or classical pneumatosis, and may be linked to steroid or indomethacin exposure, extreme prematurity, and focal defects in intestinal integrity rather than the diffuse inflammatory cascade associated with classical NEC [24]. Newer case definitions increasingly regard SIP as a distinct entity which is excluded from NEC cohorts.

Despite these advances, no single alternative definition has achieved universal adoption given the heterogeneity of NEC pathogenesis, clinical presentation and the limitations of the currently available diagnostic tools. A highly specific definition is desirable for trials, though less useful at the bedside where early diagnosis and management is important. Conversely, a more sensitive clinical definition may be acceptable for management decisions but suboptimal for research purpose. NEC may not be a single disease that can be captured completely by one fixed set of criteria. Such knowledge highlights the importance of a phenotype-based approach which explains the clinico-biological patterns leading to the disease and give guidance for diagnosis and management. A phenotype-based approach provides a stronger framework along with the operational definitions.

## 4. NEC Pathophysiology

**Figure 2:**
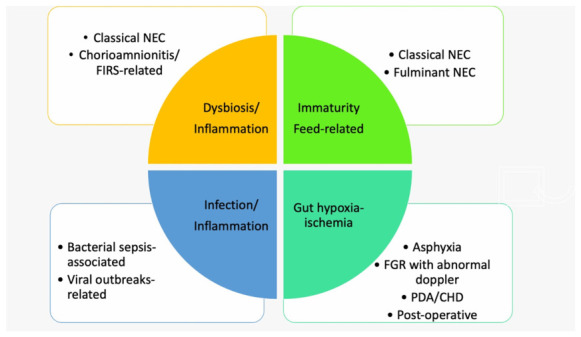
Classification of NEC into distinct phenotypes based on predominant pathophysiological mechanisms.

NEC is a multifactorial disease arising from the interplay between inherent developmental vulnerability and environmental exposures (Figure 2). Key contributing factors include intestinal immaturity, microbial dysbiosis, exaggerated inflammation, epithelial injury, and disturbances in perfusion [14,15,16,17,18,19,20,26,27,28,29].

The gastrointestinal tract of preterm infants is anatomically and functionally immature, contributing significantly to the susceptibility to NEC. The epithelial barrier, composed of tight junctions, mucus layer, and immunological defenses, is underdeveloped [30], making it more permeable to luminal bacteria and toxins.

- Tight junction proteins (e.g., claudins, occludins) are poorly expressed in preterm intestines, allowing microbial translocation into the lamina propria.
- Goblet cells and Paneth cells, critical for mucus and antimicrobial peptide production, respectively, are fewer and functionally immature, limiting mucosal defense.
- Impaired gut motility leads to stasis, promoting bacterial overgrowth.

A major mechanism in NEC pathogenesis is inappropriate activation of innate immune pathways, particularly Toll-like receptor 4 (TLR4) signaling in the immature intestinal epithelium [18,19]. Evidence suggests that excessive TLR4 activation impairs epithelial restitution, promotes enterocyte apoptosis, weakens barrier function, and exaggerates the inflammatory injury. In the preterm gut, where counter-regulatory pathways are underdeveloped, this inflammatory cascade may produce a vicious cycle of bacterial translocation, cytokine amplification, mucosal injury, and necrosis.

Microbial dysbiosis is another key component. Longitudinal studies of the preterm microbiome have identified patterns preceding NEC that include reduced microbial diversity and relative overrepresentation of Proteobacteria [20,30,31,32]. The unstable microbial ecosystem by itself does not cause NEC, but it interacts with the immature mucosa and immune system, thereby increasing inflammatory susceptibility. Human milk plays a crucial role in enhancing epithelial maturation. Components in human milk such as epidermal growth factor (EGF)**,** glutamine, and short-chain fatty acids (SCFAs) derived from microbial fermentation promote enterocyte proliferation and tight junction integrity [17,33,34].

Hypoxia and ischemia add significantly to the pathogenesis. Even mature neonates develop intestinal injury in the settings of low systemic flow, mesenteric hypoperfusion, congenital heart disease, significant patent ductus arteriosus, severe anemia, or fetal growth restriction with antenatal Doppler abnormalities [35,36,37,38,39]. In such infants, ischemia-reperfusion injury may be a major driver, perhaps interacting with feeding and inflammation. The clinical presentation may resemble classical NEC, yet differ in timing, imaging, and distribution of injury.

Inflammatory priming before or shortly after birth may also be relevant in certain infants. Exposure to chorioamnionitis, fetal inflammatory response, perinatal hypoxia, or early sepsis may sensitize the intestine and worsens subsequent intestinal injury [40,41]. These conditions could explain atypical early presentation, even before substantial feed advancement, and prominent systemic inflammation with relatively subtle abdominal signs.

These insights support a mechanistic framework in which NEC arises from overlapping pathways: microbiome-driven inflammation, immune dysregulation, and perfusion-related injury, providing a basis for phenotype-specific diagnostic and therapeutic strategies [16,17,28,29,32,42]. From a clinical perspective, prevention remains a cornerstone in reducing NEC burden. Strategies with the strongest evidence include the promotion of exclusive human milk feeding, standardized feeding protocols, careful advancement of enteral feeds, and avoidance of unnecessary antibiotic exposure [27,38,39]. Risk stratification based on gestational age, hemodynamic status, and antenatal factors may further help identify vulnerable infants and tailor preventive approaches.

## 5. NEC-Clinical Phenotypes

A phenotype-based framework recognizes that infants diagnosed with NEC do not all present in the same way, at the same time, or secondary to same pathophysiology.

### 5.1. Classical NEC

Classical NEC generally occurs in very preterm infants after initiation and advancement of enteral feeding, commonly in the second or third postnatal week, although timing varies by gestational age and ICU practices [3,43]. The process occurs secondary to a combination of intestinal immaturity, dysbiosis, and exaggerated inflammatory signaling. Typical risk factors include extreme prematurity, formula exposure or limited access to human milk, recent feed advancement, microbial dysbiosis, and episodic systemic instability. Clinically, infants often present with emesis, abdominal distension, tenderness, or visible bowel loops, followed by bloody stools or features of systemic illness. Radiographs in this phenotype usually show pneumatosis intestinalis and portal venous gas [22,23]. Formula feeding, altered colonization, and immature mucosal barrier creates an inflammatory cascade and TLR4-mediated inflammation becomes destructive [18,19]. The protective role of human milk, in contrast to formula feeding, is well established and is likely mediated through immunomodulatory, anti-inflammatory, and microbiome-modulating effects [17,33,34]. Even within this phenotype, a wide variability exists, from localized disease and recovery to the rapid progression of necrosis and perforation.

### 5.2. Early-Onset or Inflammation-Associated NEC

A subset of infants develops NEC unusually early, sometimes within the first week of life, and occasionally before significant enteral feeding is established [40,41]. In such infants, inflammation may play a more dominant role than feed-microbiome interaction alone. Maternal chorioamnionitis, fetal inflammatory response syndrome, early-onset sepsis or severe perinatal stress may lead to this phenotype. Clinically, these infants often present with systemic features out of proportion to abdominal signs and imaging findings: worsening acidosis, apnea, hypotension, escalating ventilatory need, thrombocytopenia, or poor perfusion, with only mild distension or subtle initial bowel gas abnormalities. Because pneumatosis may be absent, the clinical presentation is easily confused with septic ileus. Some infants develop later radiographic findings compatible with NEC, while others continue to pose diagnostic challenge. This phenotype does not follow the usual teaching that NEC is a post-feeding disease of the second or third week. Thus, a very immature infant with inflammatory priming may develop intestinal injury without the classical sequence assumed in Bell staging, highlighting the importance of antenatal inflammation, perinatal adaptation, and early sepsis in causing the intestinal injury.

### 5.3. Perfusion-Related NEC

In some infants with NEC, the dominant inciting factor is mesenteric hypoperfusion rather than dysbiosis or inflammation. This phenotype is seen in neonates with hemodynamically significant patent ductus arteriosus, congenital heart disease, systemic hypotension requiring inotropes, fetal growth restriction with abnormal antenatal Dopplers, or severe anemia [14,35,36,37,38,39]. In these conditions, the intestine is already compromised and chronically under-perfused, making it more susceptible to injury. Infants may show feeding intolerance, abdominal distension, acidosis, rising lactate, and poor peripheral perfusion. Pneumatosis may be absent or limited, and radiographs may show nonspecific dilated loops or a gasless abdomen. Ultrasound might show poor intestinal perfusion before radiographs become definitive [23]. Three subtypes of perfusion-related NEC have been identified.

(i) Cardiac-associated NEC: Infants with congenital heart disease, especially those with duct-dependent systemic circulation, may develop intestinal injury with NEC-like features [14,37]. The term “cardiac NEC” is often used clinically, and intestinal involvement is different from that of classical NEC with colon involved predominantly.

(ii) Fetal growth restriction (FGR)-associated intestinal injury: Growth-restricted infants exposed to abnormal antenatal Dopplers may have chronic redistribution of fetal blood flow and impaired intestinal vascular development. After birth and the initiation of enteral feeding, the immature gut exposed to chronic hypoperfusion may fail to adapt to increased metabolic demand representing a “reperfusion injury”. This condition may lead to mucosal damage, impaired motility, barrier dysfunction, and an exaggerated inflammatory response, all of which increase susceptibility to NEC [38,39].

(iii) Transfusion-associated intestinal injury: The concept of transfusion-associated NEC is controversial. Some studies attribute NEC to the preceding anemia or clinical instability rather than directly to transfusion [44]. Regardless of causality, this phenotype highlights the role of oxygen delivery and hemodynamic instability in causing intestinal injury.

### 5.4. Fulminant NEC

Fulminant NEC is characterized by rapid progression over few hours [2,3,4,45]. These infants deteriorate rapidly with severe metabolic acidosis, coagulopathy, refractory hypotension, thrombocytopenia, abdominal wall erythema or discoloration, and multiorgan dysfunction. Imaging may show extensive pneumatosis, portal venous gas, or alternatively a gasless abdomen with little warning. Surgical exploration often reveals widespread necrosis, pan-intestinal involvement, or multiple areas of ischemic injury. This phenotype highlights the limitations of progression of disease through stages, because there may be little or no clinically observed interval between “suspected” and “advanced” disease.

### 5.5. NEC in Term Infants

Although NEC is primarily a disease of prematurity, term infants can also develop this intestinal injury when morbidities like congenital heart disease, perinatal asphyxia, polycythemia, sepsis, or other stressors coexist [37,46]. Compared with classical preterm NEC, term disease more presents with identifiable precipitating conditions.

### 5.6. Postoperative or Anomaly-Associated NEC

Postoperative ileus, bowel edema, altered perfusion, stoma-related complications, and impaired motility renders NEC diagnosis following surgery or in infants with congenital anomalies challenging. Likewise, intestinal compromise in the setting of gastroschisis, omphalocele, or other anomalies may resemble NEC clinically but arise through distinct pathways.

Ultimately, a phenotypic classification is a valuable clinical tool given the variable preterm presentation, expected clinical and imaging findings, and confounding factors (Table 1).

## 6. Differential Diagnoses and Close Mimickers

The diagnosis of NEC is complicated by disorders that mimic the presentation [11,14,24,37,47,48,49,50,51,52]. Distinguishing these conditions from NEC is essential to appropriate management and prognosis.

(a) Spontaneous intestinal perforation: SIP is among the most common mimic, particularly in very immature infants [24]. It usually presents earlier than classical NEC, often within the first week of life, and results in a focal perforation rather than diffuse necrosis. In SIP, infants present with abdominal free air and abdominal distention without the classic radiological signs of pneumatosis and portal venous gas. Histologically, lesions show only focal muscularis disruption with lesser inflammation than in NEC. Risk factors include extreme prematurity, postnatal steroid exposure, and exposure to indomethacin or ibuprofen.

(b) Septic ileus: Late-onset sepsis may present with feeding intolerance, abdominal distension, apnea, lethargy, thrombocytopenia, and acidosis, all of which overlap with NEC. Dilated bowel loops can be seen in radiography but classical features like pneumatosis or pneumobilia are absent. In clinical practice, infants with suspected NEC are treated for sepsis until serial clinical evolution and imaging findings narrow the diagnosis. The distinction between septic ileus and NEC is particularly difficult in very early or inflammation-associated NEC.

(c) Cow’s milk or food protein allergy: This condition presents with bloody stools and abdominal signs in otherwise relatively stable infants [48]. Severe acidosis, thrombocytopenia, and classical radiographic findings of NEC are usually absent. The timing is often later, and improvement follows elimination of the offending protein.

(d) Viral enterocolitis: Viruses such as cytomegalovirus, rotavirus, norovirus, and enterovirus also can cause enterocolitis mimicking NEC [45]. Virally infected infants may present with distension, bloody stools, and even pneumatosis. A history of exposure and an atypical course may suggest an infectious etiology. CMV enterocolitis should be considered in very preterm infants with prolonged illness, cytopenia, and transfusion exposure.

(e) Surgical emergencies: Malrotation with midgut volvulus presents with bilious vomiting, distension, bloody stools, shock, and metabolic acidosis and may be confused with fulminant NEC [50]. Incarcerated hernia, obstructive bands, Hirschsprung-associated enterocolitis, and colonic perforation are other surgical conditions that may present similarly. Bilious vomiting, rapid hemodynamic collapse without typical imaging, and abnormal upper gastrointestinal contrast study findings can help in identifying these differentials.

(f) Others: Opioids for analgesia, anticholinergic exposure, severe respiratory disease, high continuous positive airway pressure, and electrolyte disturbances may all impair gut motility. These conditions can result in large gastric residuals and distension without intestinal inflammation. Labelling infants with these conditions as NEC unnecessarily interrupts feeding and may prolong central line exposure.

Because no single sign distinguishes NEC from all mimics, the differential diagnosis must remain active over time. Diagnostic accuracy improves when clinicians repeatedly reassess not just “how sick is the baby?” but “what process best explains the evolving findings?”

## 7. A Dynamic Diagnostic Framework for NEC

The most practical way to diagnose NEC is not using a one-time label but as a dynamic clinical process that integrates antenatal and perinatal history, serial examination, imaging, laboratory tests, and delineation of the phenotype [10,11,21,23,51]. This framework also serves as a practical bedside diagnostic and therapeutic algorithm, guiding sequential clinical decision-making.

Early diagnosis relies on recognition of evolving patterns rather than single finding, with increasing emphasis on serial clinical assessment, integration of clinical trends, and probabilistic diagnostic framework [10,11,21,53]. 

### 7.1. Step 1: Assess the Clinical Context

Gestational age, day of life, feeding exposure, type of milk, antenatal history, recent hemodynamic events, risk of infection, transfusion exposure, and congenital disease all shape diagnostic probability. A 26-week infant in week 3 on full feeds has a different profile from a 24-week infant on minimal feeds on day 4, or a term infant with congenital heart disease after surgery. Phenotyping begins at this point.

### 7.2. Step 2: Assess the Pattern of Systemic and Abdominal Findings

The next question is the determination of evolving intestinal disease as opposed to isolated feeding intolerance. Progressive abdominal distension, tenderness, discoloration, visible bowel loops, abdominal wall edema, or gross blood in stool suggest progressive intestinal disease. Systemic signs such as recurrent apnea, increasing oxygen requirement, hypotension, temperature instability, lethargy, rising lactate, thrombocytopenia, or metabolic acidosis strongly suggest an inflammatory process. No individual sign is specific, but the combination of all these signs points toward NEC.

### 7.3. Step 3: Use Serial Imaging, Not a Single Radiograph

Abdominal radiography remains the backbone of initial imaging, though its limitations must be acknowledged [22,23]. A normal or nonspecific first radiograph does not exclude NEC. Serial films may reveal evolving bowel wall gas, fixed dilated loops, portal venous gas, ascites, or free air. Serial imaging and pattern recognition is important.

Abdominal ultrasound has become an valuable adjunct [23,51]. It can assess bowel wall thickness, echogenicity, perfusion with Doppler, peristalsis, free fluid, focal collections, and bowel wall integrity. Detection of portal venous gas (PVG) using ultrasound has been shown to facilitate earlier identification of intestinal injury, sometimes preceding classical radiographic findings such as pneumatosis intestinalis [23,54]. In addition, color Doppler ultrasound enables real-time assessment of bowel perfusion, allowing earlier recognition of ischemia before abnormalities become apparent [23,51]. These modalities improve diagnostic confidence in equivocal cases and support earlier clinical decision-making. Complex ascites, absent mural perfusion, bowel wall thinning, or pneumoperitoneum may suggest advanced disease or impending perforation. The reported sensitivity and specificity of bowel ultrasound (BUS) for identifying NEC-related findings such as bowel wall perfusion abnormalities and complex ascites ranges from 85–95% and 90–96%, respectively [23,51]. Ultrasound limitations include operator dependence, yet when expertise exists, the investigation adds meaningful information relevant to diagnostic confirmation.

### 7.4. Step 4: Interpret Laboratory Tests

Common laboratory abnormalities in NEC include thrombocytopenia, metabolic acidosis, elevated lactate, neutropenia or neutrophilia, hyponatremia, coagulopathy, and increased inflammatory markers [52]. These findings help in assessing severity and offer supportive care but are not specific to NEC. Trend is more important than a single value. A falling platelet count, worsening acidosis, or increasing lactate in an infant with concerning abdominal findings suggests progression and warrants surgical consultation.

### 7.5. Step 5: Reassess the Differential at Each Stage

A dynamic framework deliberately revisits alternative diagnoses. If pneumatosis never appears, cultures become positive for bloodstream infection, and the abdomen improves rapidly with sepsis treatment, septic ileus is more likely. If the infant is extremely premature, minimally fed, and develops isolated free air early, SIP should be considered. A term infant with sudden onset bilious vomiting and shock requires the exclusion of volvulus.

### 7.6. Step 6: Use Biomarkers with Caution

A large number of biomarkers have been studied in NEC, including intestinal fatty acid-binding protein (I-FABP), fecal calprotectin, serum amyloid A, cytokine panels, claudins, and urine peptide signatures [55,56,57,58]. These are biologically appealing as they may indicate enterocyte injury, barrier disruption, and/or inflammatory activation before overt clinical or radiological findings. However, most remain limited by variable thresholds, lack of routine availability, cost, and positivity in multiple conditions. At present, biomarkers are adjuncts rather than replacements for clinical and imaging assessment.

### 7.7. Step 7: Characterize the Clinical Phenotype (Figure 3)

Instead of asking whether the child has NEC or not, we need to focus on “what intestinal process most likely explains this infant’s current presentation, our certainty regarding the condition, and which phenotype or mimic best fits the pattern?”

**Figure 3:**
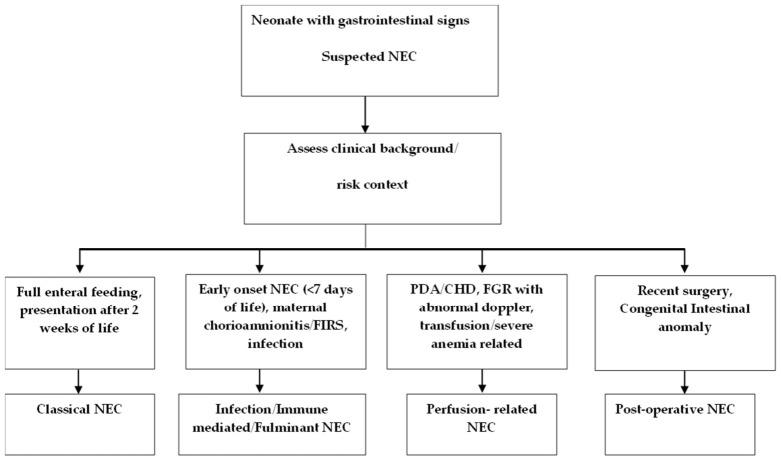
Algorithm for phenotype-based approach to suspected NEC in neonates.

## 8. Aftermath and Consequences of NEC

After the acute episode resolves, the consequences of NEC may continue for weeks to months [5,6,7,8]. Intestinal strictures, short bowel syndrome, intestinal failure, prolonged dependence on parenteral nutrition, central line-associated bloodstream infection, and intestinal failure-associated liver disease may all occur after surviving the acute phase of NEC. In some cases, long-term nutritional rehabilitation is necessary. All such complications contribute to growth failure compounded by increased metabolic demands. In addition, neurodevelopmental impairment is more frequent in infants with NEC, especially among those needing surgery [6,7,8].

## 9. Role of Predictive Models and Artificial Intelligence in Prompt Diagnosis

Predictive systems will require careful validation yet may provide an avenue important for earlier recognition of fulminant NEC. Moderate-to-high discriminative ability has been noted with predictive scoring systems and composite clinical models with area under the curve (AUC) values ranging from 0.75 to 0.88 depending on the variables included [57,58]. Artificial intelligence and machine learning may also contribute by analyzing the following trends continuously: cardiorespiratory instability, feeding patterns, lab trends, medication exposure, and imaging features [59]. Recent machine learning-based predictive models using continuous physiological and clinical data have shown excellent performance, with AUROC values approaching 0.90–0.94 for early NEC prediction [59,60,61].

## 10. Future Directions

Future progress in NEC diagnosis and prognosis will likely come from combining improved phenotyping with biological stratification [14,15,16,20,55,56,57,58]. Multi-omics approaches integrating microbiome profiles, metabolomics, transcriptomics, biomarkers, and host inflammatory signatures will help identify the dominant mechanism involved [29,32,42]. Equally important is the refinement of case definitions for research. Trials should explicitly state whether they target suspected NEC, definite NEC, surgical NEC, and/or particular phenotypes. Without this clarity, effective interventions may be missed due to inappropriate grouping of different biological phenotypes. Emerging innovations such as digital twin models, computational representations of individual patients that integrate physiological, microbiome, and clinical data, may enable individualized risk prediction and simulation of disease trajectories in NEC [29,42,61].

## 11. Conclusions

NEC remains a complex, heterogeneous condition rather than a single uniform disease. The traditional Bell staging system, though valuable for grading severity, is inadequate as a standalone diagnostic framework because early criteria are nonspecific, disease progression is not reliably linear, imaging is imperfect, and important mimics such as SIP may be misclassified. Alternative definitions are still not able to demarcate multiple biological pathways causing NEC. A phenotype-based framework offers a more clinically useful way forward. By recognizing classical feeding-associated inflammatory NEC, inflammation-associated NEC, perfusion-related NEC, fulminant NEC, term NEC, and postoperative or anomaly-associated intestinal injury, clinicians can better integrate clinical course and anticipate diagnostic challenges. The most effective diagnostic strategy is dynamic and integrative: serial clinical assessment, repeated imaging including ultrasound where available, supportive laboratory trends, and persistent reassessment of alternative diagnoses. Improving early recognition and implementing preventive strategies remain key priorities to reduce NEC-related morbidity and mortality. An improved definition is essential for improving bedside decisions, surgical timing, epidemiology, trial design, and long-term outcomes. The future of NEC diagnosis lies in moving beyond the “broken Bell” toward a phenotype-informed, and biologically grounded framework.

## Figures and Tables

**Table 1:** Clinical and pathophysiological features of necrotizing enterocolitis (NEC) phenotypes in neonates.

Endotype	Etiology	Gestation	Median Postnatal Age of Onset	Clinical Features	Radiological Findings	Histopathological Findings	Additional Testing	Outcome
Classical NEC	Dysbiosis + Immature immune response	<32 weeks	2nd–3rd week	Abdominal distension, feeding intolerance, bloody stools	Pneumatosis, portal venous gas	Transmural necrosis, inflammation, bacteria	CRP, CBC, blood culture	Variable, ~70–80% survive
**Atypical presentations in preterm neonates- Early or non-nutrition associated NEC**								
**NEC associated with infections**								
NEC and Bacterial Sepsis	Hematogenous seeding of gut	<32 weeks	Often earlier (Day 5–10)	Septic signs predominate	Similar to classical NEC	Focal/multifocal necrosis, neutrophilic infiltrate	Positive cultures, inflammatory markers	Higher mortality, Increased risk of perforation
Viral Outbreaks Triggering NEC	Enteric viruses (e.g., Norovirus, Rotavirus)	<34 weeks	1st–2nd week	Diarrhea, vomiting, apnea	Pneumatosis ± atypical pattern	Milder necrosis, lymphoid aggregates	PCR/viral panels	Variable
CMV-Associated NEC	Congenital/Perinatal CMV	Any GA, esp. <30 weeks	~1st week	NEC-like symptoms, hepatosplenomegaly	Bowel wall thickening, atypical gas pattern	CMV inclusions, necrosis	Blood CMV PCR, urine/saliva CMV PCR	Often surgical NEC
Fulminant NEC	Severe systemic inflammatory response	<28 weeks	<Day 7	Rapid progression to shock	Diffuse pneumatosis, gasless abdomen	Pan-intestinal necrosis	Blood gas, coagulation profile, cultures	High mortality (~50–80%)
**NEC related to compromised gut perfusion**								
NEC associated with PDA	Reduced mesenteric perfusion	<32 weeks	~2nd week	NEC + murmur, bounding pulses	Typical or right-sided NEC (involving predominantly the ascending colon and terminal ileum: supplied by watershed zones of the superior mesenteric artery)	Patchy ischemic necrosis	Echo for PDA	Slightly worse outcomes
NEC associated with abnormal antenatal Dopplers	Antenatal placental insufficiency	IUGR, <32 weeks	Early onset (Day 3–7)	Feed intolerance, distension	Diffuse NEC	Mucosal sloughing, ischemic changes	Antenatal doppler	Moderate survival
**Atypical presentations (Term neonates)**								
NEC associated with perinatal asphyxia	Hypoxic-ischemic injury	Term or late preterm	Day 1–3	Rapid deterioration, lactic acidosis	Free air, bowel wall edema	Ischemic necrosis without inflammation	Blood gas, Lactate, MRI brain	Often poor prognosis, Multisystem sequelae
NEC associated with congenital heart disease (CHD-NEC)	Mesenteric hypoperfusion in CHD	Term infants	Variable (Day 3–10)	Signs of NEC + cardiac symptoms	Right-sided NEC, portal gas	Ischemia-dominant	Echo, lactate	High surgical rate
NEC associated with congenital intestinal anomalies and surgery (post-operative NEC)	Post-surgical gut stress/inflammation	Term or preterm	Variable, post-op	Lethargy, ileus, fever	Difficult to detect early	Patchy necrosis, inflammation	Surgical history, cultures	Depends on underlying disease

## Data Availability

No new data were created or analyzed in this study.
